# Supplementary material for: Mixing between chemically variable primitive basalts creates and modifies crystal cargoes
Source: Nat Commun. 2021 Sep 17;12:5495. doi: 10.1038/s41467-021-25820-z (PMC8448736; doi:10.1038/s41467-021-25820-z)
Supplement: Supplementary file 2 — Supplementary Information [file 41467_2021_25820_MOESM2_ESM.pdf]

Mixing between chemically variable primitive basalts creates and modifies crystal cargoes – Supplementary information

David A. Neave[1,2]*, Philipp Beckmann[2], Harald Behrens[2] and François Holtz[2]

[1]Department of Earth and Environmental Sciences, The University of Manchester, Manchester, UK

[2]Leibniz Universität Hannover, Institut für Mineralogie, Hannover, Germany

*Email: david.neave@manchester.ac.uk

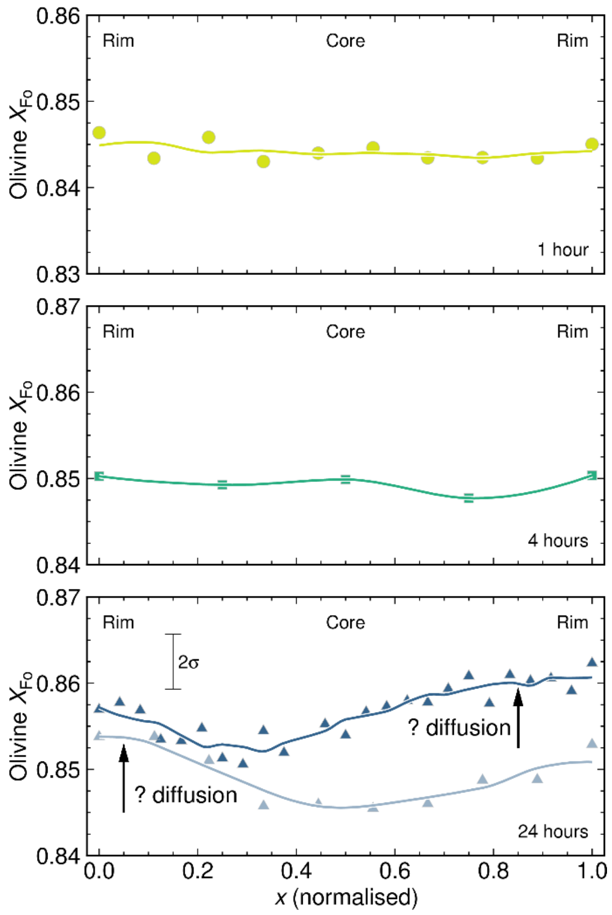

Supplementary Fig. 1 Olivine composition profiles. Olivine forsterite content [X_{Fo}, where X_{Fo} = Mg/(Mg+Fe) on molar a basis] plotted as a function of normalised distance though olivine crystals in the products of 1-, 4- and 24-hour experiments. Profiles in the products of 1- and 4-hour experiments show little variability and are consistent with little or no diffusive re-equilibration of X_{Fo}. In contrast, intracrystalline variations in X_{Fo} in the products of the 24-hour experiment are consistent with the modification of rim X_{Fo} by diffusive re-equilibration in responses to diffusively driven changes in melt FeO* contents.

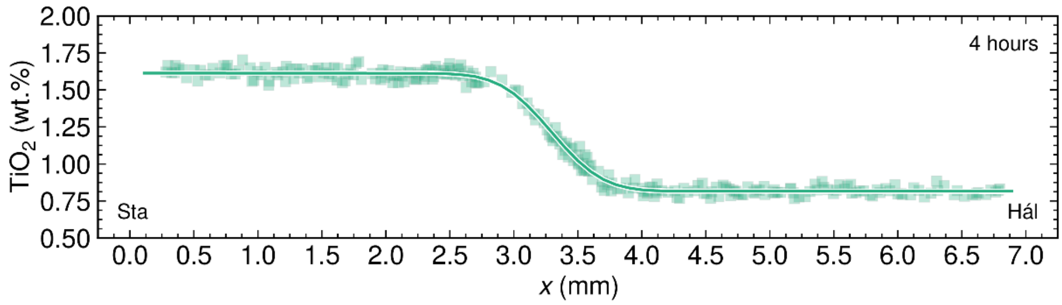

Supplementary Fig. 2 Example of an error function fit through a glass composition profile. Symbols show glass TiO_2 contents measured across the products of the 4-hour experiment. The solid line shows the error function fit used to determine an effective binary diffusion coefficient by solving Fick's 2nd Law: $C(x,t) = C_1 + \frac{C_0 - C_1}{2}\left(1 - \mathrm{erf}\left(\frac{x}{2\sqrt{Dt}}\right)\right)$, where $C(x,t)$ is the concentration in wt.% of the diffusing element C at distance x in m after time t in s, and C_0 and C_1 are the initial concentrations of the diffusing element on either side of the couple[1]. Fitting was performed by minimising the χ^2 misfit associated with the following function: $y_{est} = a\,\mathrm{erf}\big(b(x+c)\big) + d$, where y_{est} is the predicted concentration of a given oxide in wt.% and a, b, c and d are fitting parameters. The χ^2 misfit was defined as follows: $\chi^2 = \sum_{x=0}^{n}\left(\frac{(y_{obs} - y_{est})^2}{2\sigma}\right)$, where y_{obs} is the observed concentration of a given oxide in wt.% at a distance of x, y_{est} is the predicted concentration of a given oxide in wt.% at a distance of x and σ is the uncertainty associated with analyses of a given oxide in wt.%. Element diffusivities in m^2/s were then calculated using the following relationship[2]: $D = \frac{\left((1/b)^2\right)}{4t}$, where b is the fitting parameter described above. Hál and Sta refer to the Háleyjabunga and Stapafell lava analogues, respectively.

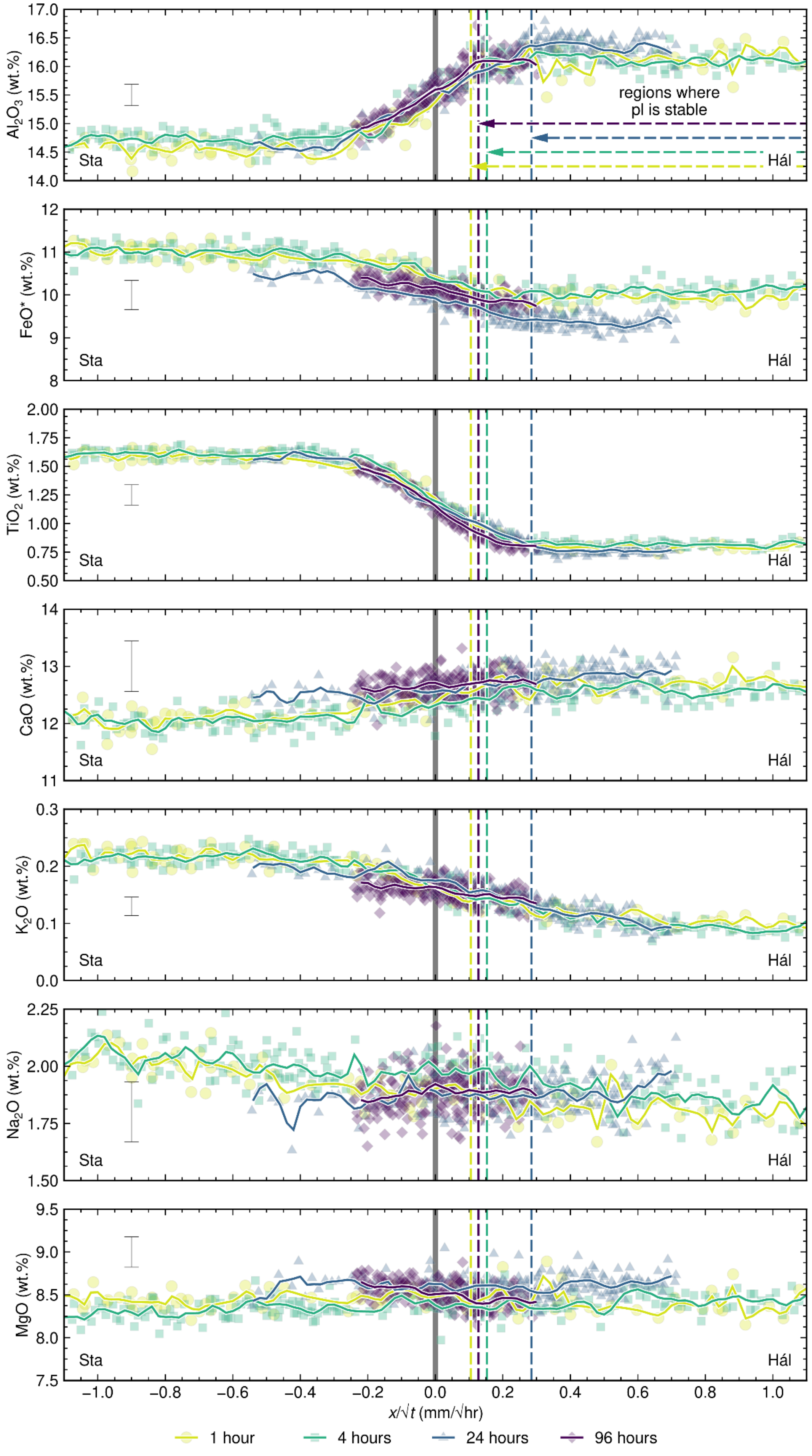

Al2O3 (wt.%)
FeO* (wt.%)
TiO2 (wt.%)
CaO (wt.%)
K2O (wt.%)
Na2O (wt.%)
MgO (wt.%)
regions where
pl is stable
Sta
Hál
x/√t (mm/√hr)
1 hour
4 hours
24 hours
96 hours

Supplementary Fig. 3 Time-normalised glass composition profiles through the products of magma-magma reaction experiments. Normalising glass composition profiles by the square root of experimental duration eliminates the effects of time on the evolution of diffusively controlled profiles, which thus collapse onto single curves for each element[3,4]. Glass composition profiles are centred on original magma-magma interfaces, which are indicated by vertical grey lines. Analyses from the products of experiments with different durations are shown with different colours and symbols. Solid lines show moving averages calculated by applying a Gaussian filter with a 0.25 mm bandwidth to raw analyses. Regions of the experimental products where plagioclase (pl) is stable are shown with dashed vertical lines and horizontal arrows. Compositions of glasses in the products of synthesis experiments are shown with grey symbols. Hál and Sta refer to the Háleyjabunga and Stapafell lava analogues, respectively. Characteristic 2σ analytical uncertainties are shown.

References cited

1. Crank, J. *The Mathematics of Diffusion*. (Clarendon Press, 1975).

2. Zhang, Y. Diffusion in minerals and melts: theoretical background. *Rev. Mineral. Geochemistry* **72**, 5–59 (2010).

3. Zhang, Y., Ni, H. & Chen, Y. Diffusion data in silicate melts. *Rev. Mineral. Geochemistry* **72**, 311–408 (2010).

4. González-Garcia, D. *et al.* Diffusive exchange of trace elements between alkaline melts: Implications for element fractionation and timescale estimations during magma mixing. *Geochim. Cosmochim. Acta* **233**, 95–114 (2018).
