# Supplementary material for: Mixing between chemically variable primitive basalts creates and modifies crystal cargoes
Source: Nat Commun. 2021 Sep 17;12:5495. doi: 10.1038/s41467-021-25820-z (PMC8448736; doi:10.1038/s41467-021-25820-z)
Supplement: Supplementary file 3 — Description of Additional Supplementary Files [file 41467_2021_25820_MOESM3_ESM.pdf]

Description of Additional Supplementary Files

File Name: Supplementary Data 1
Description: BSE map of synthesis experiment on the Háleyjabunga analogue

File Name: Supplementary Data 2
Description: BSE map of synthesis experiment on the Stapafell analogue

File Name: Supplementary Data 3
Description: BSE map of the 1-hour mixing experiment.

File Name: Supplementary Data 4
Description: BSE map of the 4-hour mixing experiment.

File Name: Supplementary Data 5
Description: BSE map of the 24-hour mixing experiment.

File Name: Supplementary Data 6
Description: BSE map of the 96-hour mixing experiment.

File Name: Supplementary Data 7
Description: : EMPA data from glasses and minerals, typical analyses of secondary standards, summaries of experimental conditions and estimated binary diffusion coefficients
