# Supplementary figures and images for: Mixing between chemically variable primitive basalts creates and modifies crystal cargoes
Source: Nat Commun. 2021 Sep 17;12:5495. doi: 10.1038/s41467-021-25820-z (PMC8448736; doi:10.1038/s41467-021-25820-z)

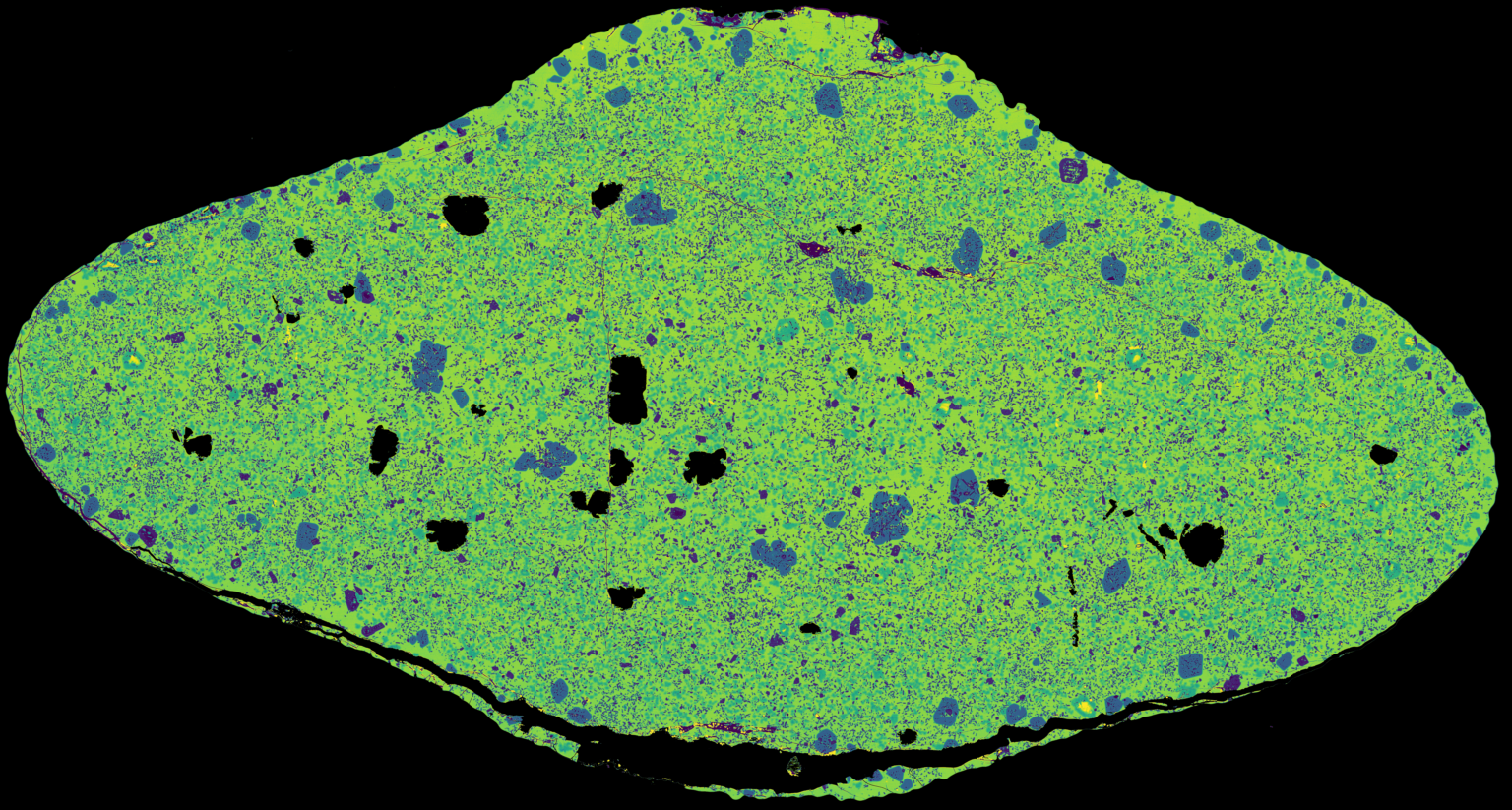

Supplement: Supplementary file 4 — Supplementary Data 1-7 [file 41467_2021_25820_MOESM4_ESM.zip › supplementary_data_1.png]

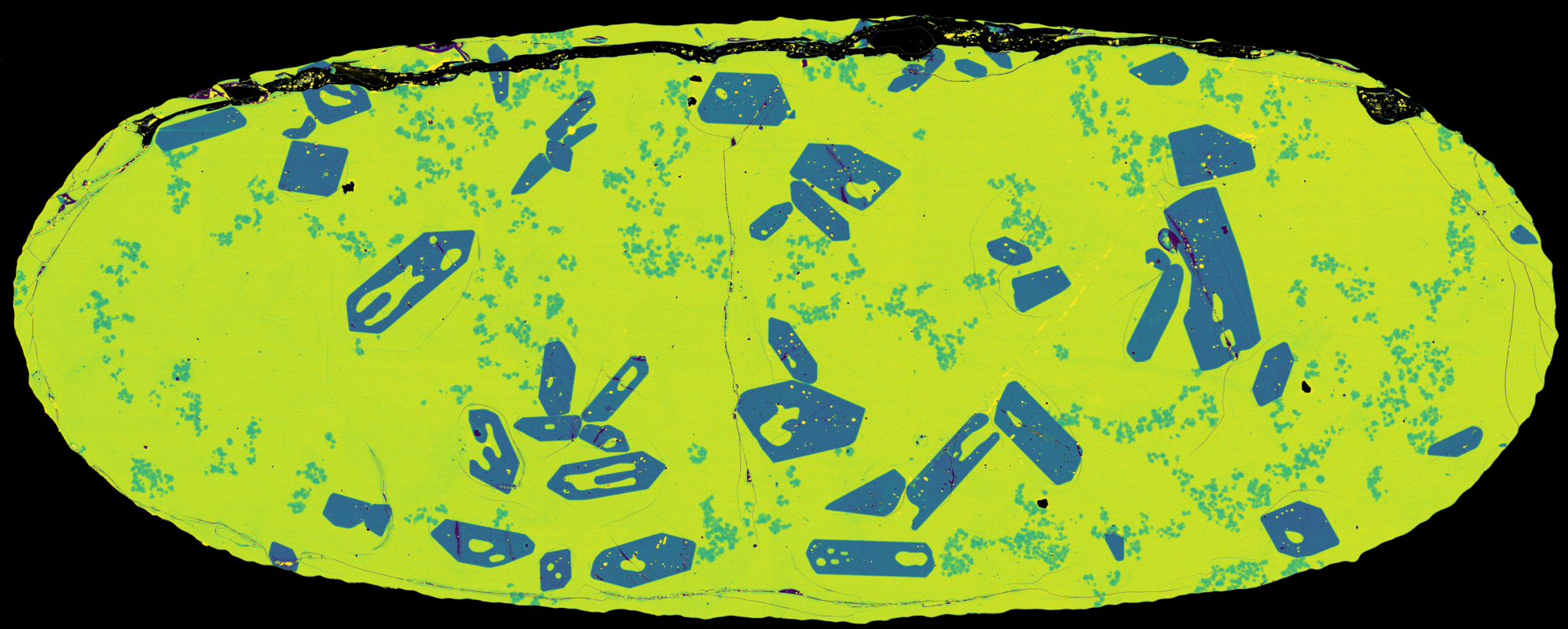

Supplement: Supplementary file 4 — Supplementary Data 1-7 [file 41467_2021_25820_MOESM4_ESM.zip › supplementary_data_2.png]

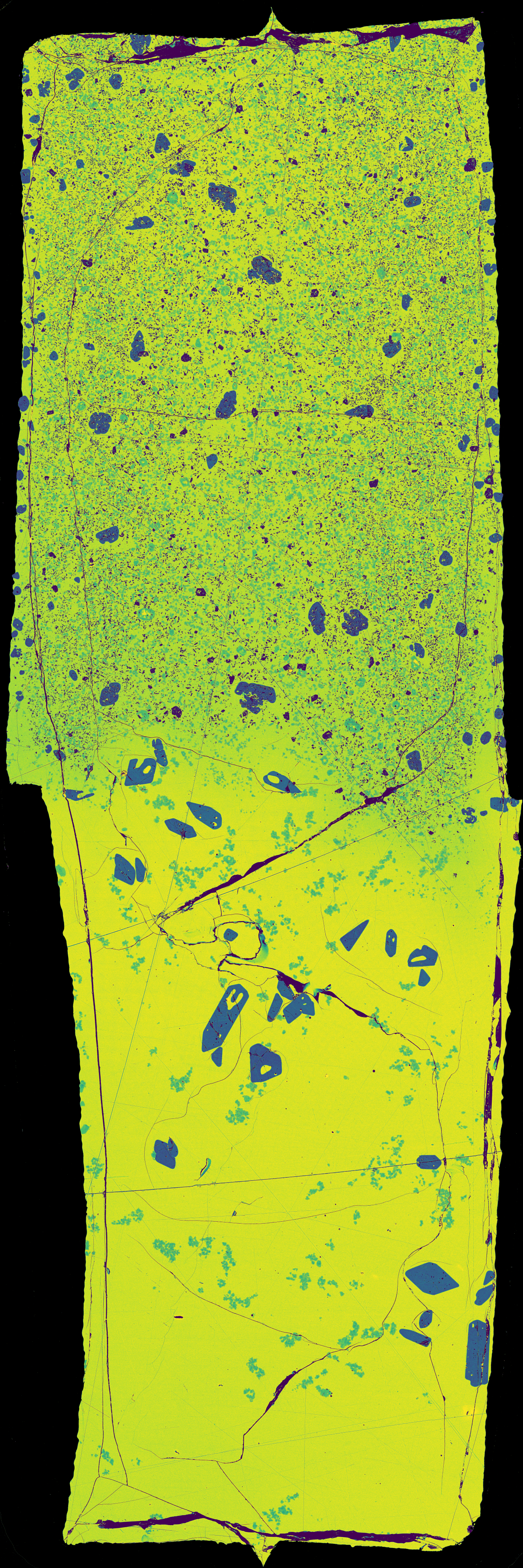

Supplement: Supplementary file 4 — Supplementary Data 1-7 [file 41467_2021_25820_MOESM4_ESM.zip › supplementary_data_3.png]

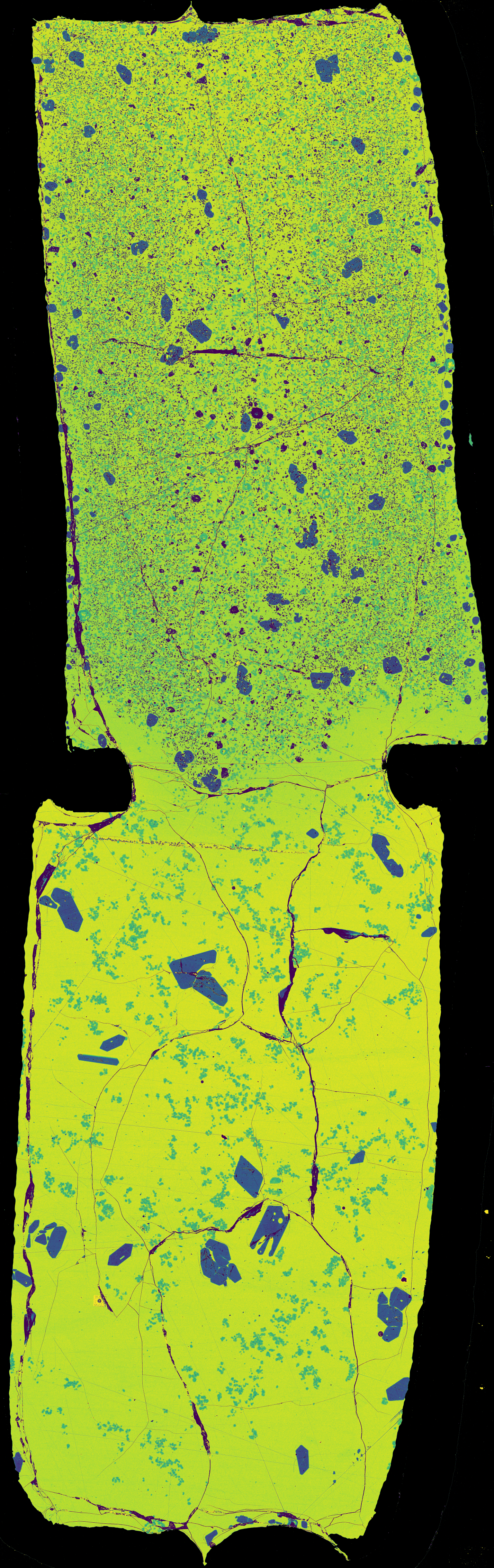

Supplement: Supplementary file 4 — Supplementary Data 1-7 [file 41467_2021_25820_MOESM4_ESM.zip › supplementary_data_4.png]

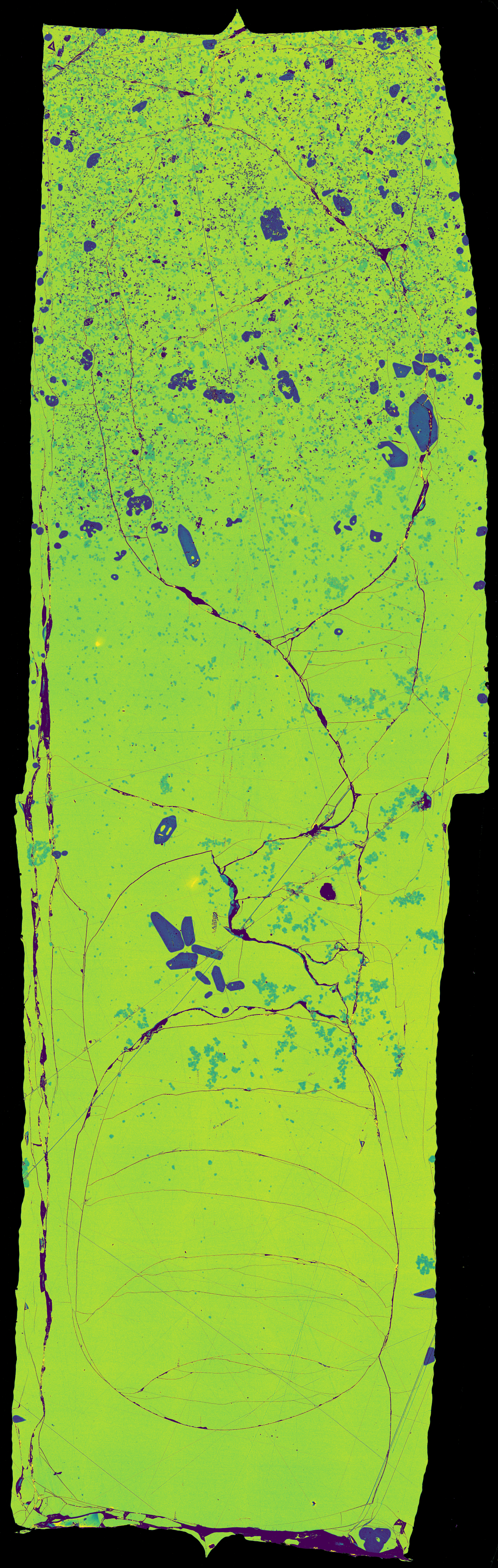

Supplement: Supplementary file 4 — Supplementary Data 1-7 [file 41467_2021_25820_MOESM4_ESM.zip › supplementary_data_5.png]

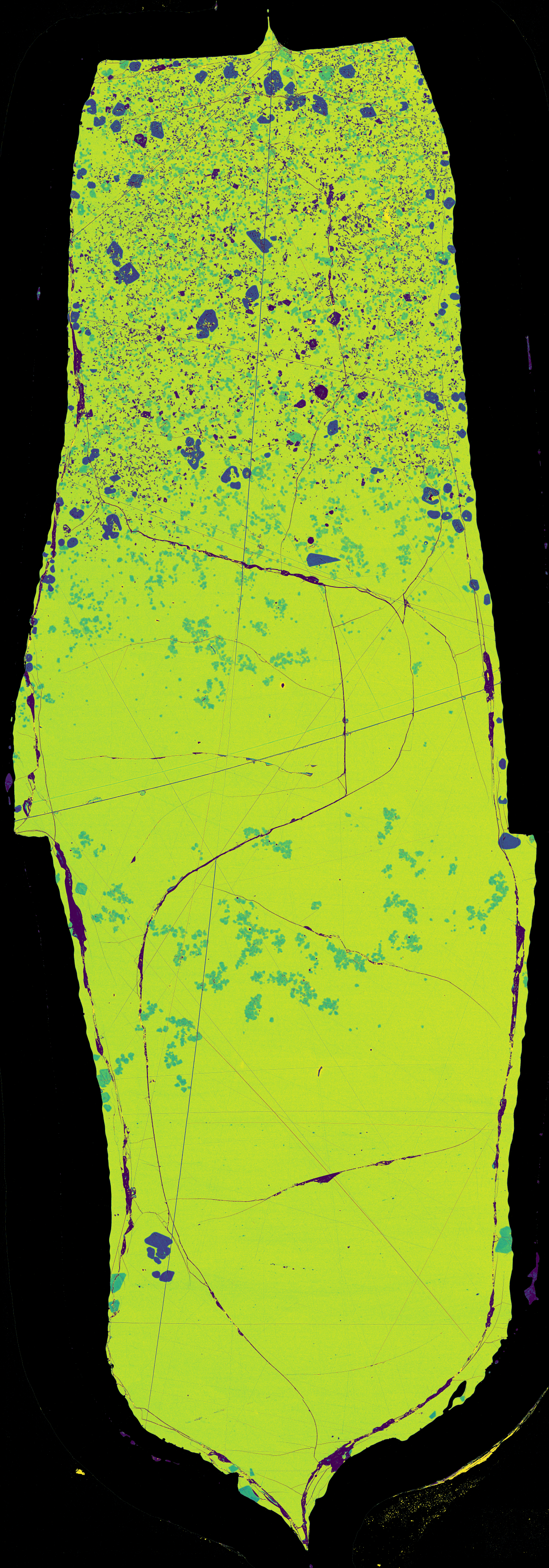

Supplement: Supplementary file 4 — Supplementary Data 1-7 [file 41467_2021_25820_MOESM4_ESM.zip › supplementary_data_6.png]
